# Analysis of codon usage patterns of the chloroplast genome in *Delphinium grandiflorum* L. reveals a preference for AT-ending codons as a result of major selection constraints

**DOI:** 10.7717/peerj.10787

**Published:** 2021-01-18

**Authors:** Huirong Duan, Qian Zhang, Chunmei Wang, Fang Li, Fuping Tian, Yuan Lu, Yu Hu, Hongshan Yang, Guangxin Cui

**Affiliations:** 1 Chinese Academy of Agricultural Sciences Lanzhou Institute of Husbandry and Pharmaceutical Science, Lanzhou, China; 2 Institute of Grassland Science, Chinese Academy of Agricultural Sciences, Hohhot, China

**Keywords:** *Delphinium grandiflorum* L., Chloroplast genome, Synonymous codon usage bias, Evolutionary forces

## Abstract

**Background:**

Codon usage bias analysis is a suitable strategy for identifying the principal evolutionary driving forces in different organisms. *Delphinium grandiflorum* L. is a perennial herb with high economic value and typical biological characteristics. Evolutionary analysis of *D. grandiflorum* can provide a rich resource of genetic information for developing hybridization resources of the genus *Delphinium*.

**Methods:**

Synonymous codon usage (SCU) and related indices of 51 coding sequences from the *D. grandiflorum* chloroplast (cp) genome were calculated using Codon W, Cups of EMBOSS, SPSS and Microsoft Excel. Multivariate statistical analysis combined by principal component analysis (PCA), correspondence analysis (COA), PR2-plot mapping analysis and ENC plot analysis was then conducted to explore the factors affecting the usage of synonymous codons.

**Results:**

The SCU bias of *D. grandiflorum* was weak and codons preferred A/T ending. A SCU imbalance between A/T and G/C at the third base position was revealed by PR2-plot mapping analysis. A total of eight codons were identified as the optimal codons. The PCA and COA results indicated that base composition (GC content, GC_3_ content) and gene expression were important for SCU bias. A majority of genes were distributed below the expected curve from the ENC plot analysis and up the standard curve by neutrality plot analysis. Our results showed that with the exception of notable mutation pressure effects, the majority of genetic evolution in the *D. grandiflorum* cp genome might be driven by natural selection.

**Discussions:**

Our results provide a theoretical foundation for elucidating the genetic architecture and mechanisms of *D. grandiflorum*, and contribute to enriching *D. grandiflorum* genetic resources.

## Introduction

The codon is crucial in the process of genetic information transmission, and is the most fundamental step in biological activities (Powell & Moriyama, 1997; Chen et al., 2014). The accurate identification of codons encoding different amino acids is key to ensuring the correct expression of genetic information (Morton, Sorhannus & Fox, 2002; Sau et al., 2006). Most of the amino acids (except methionine (Met) and tryptophan (Trp)) are encoded by two to six synonymous codons (Guan et al., 2018). The choices of synonymous codons in different plant genomes are non-random, which is known as synonymous codon usage (SCU) bias (Wright, 1990). SCU bias reflects a mutation-selection balance, which can be affected by mutation pressure, natural selection, and genetic drift in a population (Bulmer, 1991; Eyre-Walker, 1991). Therefore, understanding the SCU bias can reveal the effects of long-term evolution on plant genomes.

The possible evolutionary forces based on codon usage patterns have been investigated in the genomes of numerous organisms. Generally, codon usage biases in microbes are driven by mutation pressure, such as in *Xanthophyllomyces dendrorhous* and *Escherichia coli* (Baeza et al., 2015; Boël et al., 2016). For invertebrate animals, codon usage bias is mainly driven by selection constraints, as exemplified in *Bemisia tabaci* and *Hirudinaria manillensis* (Sharma, Chakraborty & Uddin, 2014). Additionally, in plant species, codon usage bias seems to prefer a balance of mutation pressure and selection constraints (Zhang et al., 2018a; Zhang et al., 2018b; Zhang et al., 2018c; Liu et al., 2010). In the rice genome, the heterogeneity of codon usage patterns reflects a balance between a directional mutational bias and negative selection (Wang & Hickey, 2007). The codon usage bias in the *Porphyra umbilicalis* chloroplast (cp) genome is influenced by natural selection, mutation pressure, and nucleotide composition (Li et al., 2019). Moreover, codon usage bias results from Wang et al. (2018) indicated that translation selection has a more dominant role than mutation pressure in four cotton species. These studies indicate that complex evolutionary factors vary in different organisms, and analyzing codon usage bias can provide suitable strategies for identifying the principal driving forces. *Delphinium grandiflorum* L. (Ranunculaceae, *Delphinium*), a perennial herb with a blue flower, is mainly distributed in Mongolia, Siberia, and the Northwest of China (Chen et al., 2017). Owing to its high contents of two novel diterpenoid alkaloids, namely, grandiflodines A and B, *D. grandiflorum* is cultivated as a medicinal plant for toothache treatment and as a native pesticide (Zhang, Li & He, 2012). Furthermore, ovule culture is applied in *D. grandiflorum* to avoid hybrid embryos from aborting. For example, new interspecific hybrid plants (*D. grandiflorum* × *D. nudicaule, D. grandiflorum* × *D. cardinal*) are successfully selected with the intermediate flower color between the parents (Honda, Tsutsui & Hosokawa, 1999). Thus, *D. grandiflorum* is of great biological significance, and evolutionary analysis of *D. grandiflorum* can provide a rich resource of genetic information for developing hybridization resources for the genus *Delphinium*.

The chloroplast is a photosynthetic organelle in plant cells that plays crucial roles in photosynthesis and metabolite biosynthesis, for example, the synthesis of amino acids, starch, fatty acids, and pigments (Wicke et al., 2011). Compared to the mitochondrial genome and nuclear genome, the complete cp genome, which possesses many characteristics, including a small size, simple and highly conserved structure, single parental inheritance, and haploid nature, is widely applied in species identification, phylogenetic analysis, and adaptive evolutionary analysis (Raubeson et al., 2007). Codon usage in many plant species, such as *Hemiptelea davidii, Haberlea rhodopensis, Medicago sativa*, and so forth, has been investigated extensively based on the cp genome database (Liu et al., 2020; Ivanova et al., 2017; Tao et al., 2017). The cp genome of *D. grandiflorum* has been assembled and characterized using Illumina sequencing platform, it was 157,339 bp in length, which contained a pair of inverted repeated regions (52,304 bp), a large single copy region (88,098 bp) and a small single copy region (16,937 bp) (Duan et al., 2020). However, the SCU bias of *D. grandiflorum* cp genome has not been investigated.

In this study, we analyzed the codon bias and related indices of *D. grandiflorum* cp DNA, and then used multivariate statistical analysis to determine the general evolutionary driving factors. These results improve our understanding of the genetic architecture of *D. grandiflorum*, and also contribute to enriching the genetic resources and conservation of *D. grandiflorum* species.

## Materials & Methods

### Sequence data

A total of 117 genes were obtained from the *D. grandiflorum* cp genome (Genbank accession number: MN556604), and the sequence information is shown in Table S1 (Duan et al., 2020). After filtering the repeated sequences and genes with sequence length <300 bp using an in-house Python script (Sanner, 1999), ORFfinder (http://www.geneinfinity.org/sms/sms_orffinder.html) was used to distinguish and filter out non-coding regions of the remaining genes (Guan et al., 2018). Finally, a total of 51 qualified CDSs (complete coding sequence) were retained for subsequent analysis.

### Codon usage bias and related indices analysis

A number of the codon usage indicators were estimated via the program codon W version 1.3 (https://sourceforge.net/projects/codonw/), including the relative synonymous codon usage value (RSCU), the effective number of codons (ENC), G + C content of the gene (GC), the frequency of the nucleotides G + C at the 3rd position of synonymous codons (GC_3s_), and the base compositions (A_3s_, T_3s_, G_3s_, and C_3s_) (Zhang et al., 2018a; Zhang et al., 2018b; Zhang et al., 2018c). The RSCU value and ENC value were used together to describe codon usage patterns. The G+C content at the 1st, 2nd, 3rd of codons (GC_1_, GC_2_, GC_3_) and the average GC content of the 1st and 2nd (GC_12_) were determined by the Cusp function from EMBOSS (http://imed.med.ucm.es/cgi-bin/emboss.pl?_action=input_app=cusp).

### Identification of the optimal codon

According to the RSCU values, the synonymous codons with the highest frequencies, accompanied by the largest RSCU values, were identified (Yu et al., 2012). Using ENC analysis as a preference standard, the 51 sequences of *D. grandiflorum* were ordered, and 5% of the dataset with high bias (ENC value was less than 30) and low bias (ENC value was larger than 55) were selected (Cui et al., 2020). The sequences with high bias and low bias were recognized as highly and lowly expressed genes, respectively, as a result of codon bias and were positively correlated with gene expression level (Li et al., 2016). Highly expressed codons, were defined as those codons that occurred significantly more often in highly expressed genes relative to their frequency in lowly expressed genes, which was reflected by ΔRSCU. The ΔRSCU of each codon was calculated following the formula of ΔRSCU = RSCU (high bias) - RSCU (low bias) (Wang et al., 2019). Finally, the optimal codon of the gene was speculated as the codon with both the highest RSCU value and the largest ΔRSCU (Sharp & Li, 1986).

### Multivariate statistical analysis

Principal component analysis (PCA) was used as a dimensionality reduction tool to reduce the data complexity in CodonW, with the principal components used to explore the codon usage variation among genes (Greenacre, 1984). PCA was performed on the RSCU values, the data were plotted in a 59-dimensional space of different axes, and the 59-dimensional space was based on the 59 triplet nucleotide codons (ATG encoding Met and TGG encoding Trp were excluded) (Gupta & Ghosh, 2001). Finally, the most prominent axes with important implications for codon usage variation were revealed (Choudhury, Uddin & Chakraborty, 2017).

Correspondence analysis (COA) was used to compare two or more categories of variable data, and provide visual results for the major changes in the trends of codon usage and genes (Choudhury, Uddin & Chakraborty, 2017). The relationship between prominent axes and codons, prominent axes and GC content, and prominent axes and genes were visualized in scatter plots.

Parity rule 2 (PR2) plot mapping analysis was used to show the relationship of the values A_3_/(A_3_ + T_3_) and G_3_/(G_3_ + C_3_) related to codons and four-degenerate synonymous-codon amino acids (alanine, glycine, proline, threonine, valine, arginine [CGA, CGU, CGG, and CGC], leucine [CUA, CUU, CUG, and CUC] and serine [UCA, UCU, UCG, and UCC]), then the data were distributed into four quadrants in a scatter diagram (Sueoka, 1995; Sueoka, 1999).

ENC-plot mapping analysis was employed to analyze and determine the crucial factors influencing the codon usage bias. The ENC plot reflects the relationship of the ENC values against the GC_3S_ values. The standard curve shows the optimal functional relation between ENC and GC_3s_ (Gupta, Bhattacharyya & Ghosh, 2004).

Neutrality plot mapping analysis was used to analyze the relationship of the GC_12_ values and GC_3_ values of all the genes. In the neutral graph, the value of GC_12_ was used as a vertical coordinate, and the value of GC_3_ was used as the horizontal axis (Wei et al., 2014).

### Statistical analysis

Correlation analysis among many important indices was implemented in SPSS 16.0 software (SPSS Inc., Chicago, US) with the Spearman’s test (two-tailed). The graphs were depicted in Microsoft EXCEL 2016 (Microsoft Corporation, Redmond, WA, US).

## Results

### Nucleotide composition between codon positions in the *D. grandiflorum* cp genome

**Figure 1:**
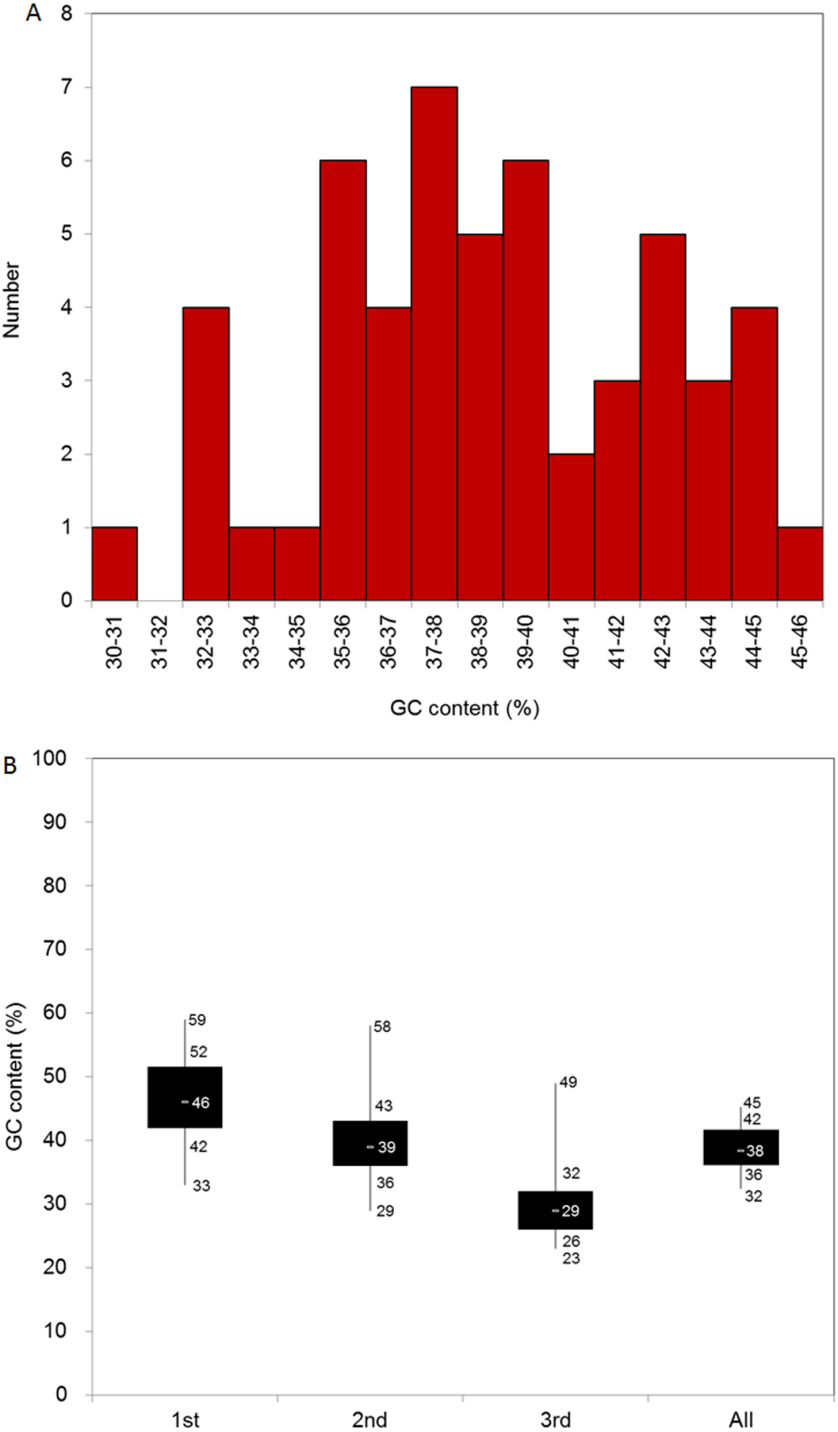
Base composition of *D. grandiflorum* cp genome. (A) Distribution of genes with different GC contents; (B) Box plot of GC contents variation in different codon positions (1st, 2nd, 3rd and all (overall cp genome)). The numbers on the box plot from top to bottom represent GC content of the maximum, upper quartile (75%), middle quartile (50%), lower quartile (25%), and minimum, respectively.

We identified 51 CDSs longer than 300 bp, and the average length of the CDSs was 1212.6 bp. In general, the four nucleotides were unevenly represented in the 51 CDSs. Thymine (T) was the most represented (31%), adenine (A) was the second-most represented (30%), cytosine (C) and guanine (G) were less represented (18% and 21%, respectively), and the average GC content of the CDSs was 39%. To better evaluate the nucleotide base composition in *D. grandiflorum*, we summarized the CDS numbers with different GC content levels, and all CDSs contained 30–46% GC content (Fig. 1A). We further divided the GC content range into three parts and analyzed the number of CDSs attributable to each part. The 35–40% part contained the most CDSs (the total number was 28), followed sequentially by the 30–35% and 40–46% intervals.

We also summed the GC content at different codon positions (1st, 2nd, and 3rd) in the CDSs. The composition at the 2nd codon position was similar to that of the overall nucleotide composition. The average GC content and the range between the upper and lower quartiles in the 1st codon position were the highest, and accordingly, the corresponding data in the 3rd codon position were the lowest (Fig. 1B).

### The codon usage pattern of the *D. grandiflorum* cp genome

The amino acids number of 51 genes ranged between 101 and 2,138 with an average of 404. We identified a total of 61 synonymous codons (stop codons were excluded), among which 31 were more frequently represented with an RSCU value ≥ 1 (Table 1). The codon TTA encoding Leu exhibited the highest RSCU value of 1.88. The above 31 codons with different end bases were divided into three classes, and the number of codons ending with T, A, and G was 16, 12, and 3, respectively, thus suggesting that the genes from the *D. grandiflorum* cp genome preferred codons with AT-endings, especially those ending with T. Moreover, we focused on the preferred and weak preferred codons, mainly emphasizing codons with extremely high (>1.5) and low RSCUs (<0.5). We found that codons such as ACT, TAT, CAA, and GGA were highly preferred and codons such as CTC, AAC, CTG, and CAG were less preferred in the CDSs. The two distinct patterns deviated from the neutral RSCU value of 1, further indicating that codons preferred an ending with A/T.

**Table 1:** Codon usage and high frequency used codons in *D. grandiflorum* cp genome. The highest frequency used codons (the largest RSCU value) are in bold. RSCU: the relative synonymous codon usage value.

Amino acid	Condon	Number	RSCU	Amino acid	Condon	Number	RSCU
Ala (A)	**GCT**	**482**	**1.71**	Asn (N)	**AAT**	**775**	**1.57**
	GCC	186	0.66		AAC	214	0.43
	GCA	326	1.15	Pro (P)	**CCT**	**331**	**1.51**
	GCG	136	0.48		CCC	166	0.76
Cys (C)	**TGT**	**170**	**1.47**		CCA	257	1.18
	TGC	61	0.53		CCG	120	0.55
Asp (D)	**GAT**	**677**	**1.57**	Gln (Q)	**CAA**	**550**	**1.52**
	GAC	183	0.43		CAG	173	0.48
Glu (E)	**GAA**	**820**	**1.48**	Arg (R)	CGT	280	1.38
	GAG	290	0.52		CGC	76	0.37
Phe (F)	**TTT**	**749**	**1.32**		CGA	276	1.36
	TTC	386	0.68		CGG	88	0.43
Gly (G)	GGT	482	1.34		**AGA**	**360**	**1.77**
	GGC	162	0.45		AGG	139	0.68
	**GGA**	**552**	**1.54**	Ser (S)	**TCT**	**423**	**1.65**
	GGG	241	0.67		TCC	252	0.99
His (H)	**CAT**	**386**	**1.48**		TCA	311	1.22
	CAC	135	0.52		TCG	160	0.63
Ile (I)	**ATT**	**874**	**1.48**		AGT	299	1.17
	ATC	344	0.58		AGC	90	0.35
	ATA	558	0.94	Thr (T)	**ACT**	**421**	**1.59**
Lys (K)	**AAA**	**763**	**1.47**		ACC	194	0.73
	AAG	274	0.53		ACA	329	1.24
Leu (L)	**TTA**	**673**	**1.88**		ACG	116	0.44
	TTG	458	1.28	Val (V)	GTT	421	1.51
	CTT	456	1.28		GTC	114	0.41
	CTC	135	0.38		**GTA**	**430**	**1.54**
	CTA	288	0.81		GTG	152	0.54
	CTG	135	0.38	Tyr (Y)	**TAT**	**617**	**1.59**
Met (M)	ATG	500	1		TAC	157	0.41
Trp (W)	TGG	386	1				

The average content of GC, GC_1_, GC_2_, and GC_3_ of the CDSs from the *D. grandiflorum* was calculated (Table 2). The ENC values of the different genes varied from 37.11 to 61.00, the average of which was 48.12, displaying different trends between the genes. Strong and weak SCU biases are typically distinguished by the ENC value with 35, and all of the ENC values of the genes in this study were greater than 35, suggesting a weak codon bias (Song et al., 2018).

**Table 2:** Indices of codon usage of 51 genes from the cp genome of *D. grandiflorum*.

**Gene**	**GC**	**GC**_**3**_	**GC**_**3S**_	**ENC**	**Gene**	**GC**	**GC**_**3**_	**GC**_**3S**_	**ENC**
*accD*	0.36	0.31	0.28	50.99	*psbA*	0.43	0.34	0.30	40.89
*atpA*	0.42	0.31	0.29	49.59	*psbB*	0.43	0.30	0.25	47.36
*atpB*	0.42	0.29	0.27	47.81	*psbC*	0.45	0.33	0.30	46.17
*atpE*	0.39	0.27	0.25	47.65	*psbD*	0.44	0.35	0.30	46.28
*atpF*	0.38	0.34	0.32	44.90	*rbcL*	0.44	0.31	0.29	49.45
*atpI*	0.38	0.27	0.24	46.31	*rpl14*	0.39	0.26	0.24	49.88
*ccsA*	0.32	0.25	0.20	46.36	*rpl16*	0.44	0.27	0.21	41.65
*cemA*	0.32	0.31	0.27	56.82	*rpl20*	0.37	0.29	0.26	43.10
*clpP*	0.45	0.34	0.31	55.82	*rpl22*	0.36	0.30	0.24	47.84
*matK*	0.31	0.26	0.23	47.53	*rpoA*	0.36	0.29	0.26	48.30
*ndhA*	0.36	0.25	0.22	42.71	*rpoB*	0.40	0.30	0.28	50.41
*ndhB*	0.37	0.32	0.27	47.37	*rpoC1*	0.38	0.28	0.25	48.97
*ndhC*	0.36	0.26	0.20	41.24	*rpoC2*	0.38	0.29	0.27	50.39
*ndhD*	0.36	0.29	0.24	48.99	*rps11*	0.45	0.26	0.22	50.83
*ndhE*	0.34	0.23	0.20	52.21	*rps14*	0.41	0.32	0.29	40.28
*ndhF*	0.33	0.24	0.20	44.71	*rps18*	0.35	0.24	0.21	37.11
*ndhG*	0.36	0.26	0.22	43.72	*rps2*	0.38	0.26	0.23	45.79
*ndhH*	0.39	0.30	0.25	51.44	*rps3*	0.36	0.26	0.24	48.96
*ndhI*	0.37	0.28	0.26	50.38	*rps4*	0.39	0.27	0.26	51.15
*ndhJ*	0.41	0.35	0.30	57.52	*rps7*	0.41	0.24	0.21	46.08
*ndhK*	0.40	0.30	0.27	51.77	*rps8*	0.37	0.25	0.22	42.77
*petA*	0.39	0.32	0.30	50.42	*ycf1*	0.32	0.29	0.25	49.67
*petB*	0.43	0.36	0.30	45.84	*ycf2*	0.38	0.37	0.34	53.44
*petD*	0.38	0.24	0.21	40.60	*ycf3*	0.40	0.49	0.46	61.00
*psaA*	0.44	0.35	0.30	50.76	*ycf4*	0.40	0.34	0.31	51.36
*psaB*	0.42	0.34	0.29	51.35	Average	0.39	0.30	0.26	48.12

A total of 18 codons with the largest RSCU value based on each amino acid were identified as high frequency synonymous codons (Table 1). Twenty-three codons were identified as highly expressed codons (Table 3). Eight codons with a high frequency as well as high expression, including GCT, GAT, TTT, ATT, AAA, TCT, ACT, and TAT, were identified as optimal codons, of which, seven ended with T and only one ended with A, further confirming that the codons ending with C and G were lacking preference in the *D. grandiflorum* cp genome.

**Table 3:** The codons statistics with high and low expression genes of the *D. grandiflorum* cp genome.

Amino acid	Codon	High expressed gene		Low expressed gene		ΔRSCU	Amino acid	Codon	High expressed gene		Low expressed gene		ΔRSCU
		Frequency	RSCU	Frequency	RSCU				Frequency	RSCU	Frequency	RSCU	
Ala (A)	GCT^*^	4.00	1.45	4.00	1.00	0.45	Asn (N)	AAT	4.00	1.33	2.00	1.33	0.00
	GCC	1.00	0.36	3.00	0.75	−0.39		AAC	2.00	0.67	1.00	0.67	0.00
	GCA	4.00	1.45	6.00	1.50	−0.05	Pro (P)	CCT	4.00	1.60	5.00	1.54	0.06
	GCG	2.00	0.73	3.00	0.75	−0.02		CCC	1.00	0.40	2.00	0.62	−0.22
Cys (C)	TGT	0.00	0.00	3.00	2.00	−2.00		CCA^*^	5.00	2.00	2.00	0.62	1.38
	TGC	0.00	0.00	0.00	0.00	0.00		CCG	0.00	0.00	4.00	1.23	−1.23
Asp (D)	GAT^*^	7.00	2.00	0.00	0.00	2.00	Gln (Q)	CAA^*^	6.00	2.00	4.00	1.00	1.00
	GAC	0.00	0.00	3.00	2.00	−2.00		CAG	0.00	0.00	4.00	1.00	−1.00
Glu (E)	GAA	8.00	1.33	12.00	1.85	−0.52	Arg (R)	CGT	4.00	1.20	9.00	1.59	−0.39
	GAG^*^	4.00	0.67	1.00	0.15	0.52		CGC	0.00	0.00	1.00	0.18	−0.18
Phe (F)	TTT^*^	13.00	1.53	4.00	1.33	0.20		CGA^*^	9.00	2.70	9.00	1.59	1.11
	TTC	4.00	0.47	2.00	0.67	−0.20		CGG	0.00	0.00	0.00	0.00	0.00
Gly (G)	GGC^*^	1.00	0.36	1.00	0.21	0.15		AGA	6.00	1.80	11.00	1.94	−0.14
	GGA	4.00	1.45	9.00	1.89	−0.44		AGG	1.00	0.30	4.00	0.71	−0.41
	GGG	2.00	0.73	4.00	0.84	−0.11	Ser (S)	TCT^*^	6.00	1.89	6.00	1.71	0.18
	GGT^*^	4.00	1.45	5.00	1.05	0.40		TCC^*^	3.00	0.95	3.00	0.86	0.09
His (H)	CAT	0.00	0.00	5.00	1.67	−1.67		TCA	1.00	0.32	4.00	1.14	−0.82
	CAC^*^	1.00	2.00	1.00	0.33	1.67		TCG^*^	3.00	0.95	3.00	0.86	0.09
Ile (I)	ATT^*^	14.00	1.83	8.00	1.41	0.42		AGT^*^	6.00	1.89	5.00	1.43	0.46
	ATC	2.00	0.26	2.00	0.35	−0.09		AGC	0.00	0.00	0.00	0.00	0.00
	ATA	7.00	0.91	7.00	1.24	−0.33	Thr (T)	ACT^*^	5.00	2.50	1.00	0.40	2.10
Lys (K)	AAA^*^	9.00	1.64	14.00	1.56	0.08		ACC	0.00	0.00	3.00	1.20	−1.20
	AAG	2.00	0.36	4.00	0.44	−0.08		ACA	0.00	0.00	6.00	2.40	−2.40
Leu (L)	TTA	8.00	1.78	4.00	1.71	0.07		ACG^*^	3.00	1.50	0.00	0.00	1.50
	TTG	8.00	1.78	4.00	1.71	0.07	Val (V)	GTT^*^	9.00	2.57	4.00	1.45	1.12
	CTT	7.00	1.56	6.00	2.57	−1.01		GTC	0.00	0.00	1.00	0.36	−0.36
	CTC	0.00	0.00	0.00	0.00	0.00		GTA	3.00	0.86	6.00	2.18	−1.32
	CTA^*^	3.00	0.67	0.00	0.00	0.67		GTG^*^	2.00	0.57	0.00	0.00	0.57
	CTG^*^	1.00	0.22	0.00	0.00	0.22	Trp (W)	TGG	5.00	1.00	5.00	1.00	0.00
Met (M)	ATG	6.00	1.00	8.00	1.00	0.00	Tyr (Y)	TAT^*^	7.00	2.00	6.00	1.71	0.29
								TAC	0.00	0.00	1.00	0.29	−0.29

**Notes.**

RSCU: the relative synonymous codon usage value.

* indicates the high expression codons (ΔRSCU > 0.08).

### PCA analysis

The 51 CDSs of the *D. grandiflorum* cp genome were analyzed using PCA analysis, and were distributed in 50 dimensional axes. The contribution of 40 axes was calculated, and the gene variations from the four major axes (Axis 1 to Axis 4) accounted for 35.5% of the total axis variation. Axis 1 and Axis 2 explained 10.71% and 8.96% of the total variation, while Axis 3 and Axis 4 explained 8.36% and 7.47% of that the variation, respectively.

### COA analysis

**Figure 2:**
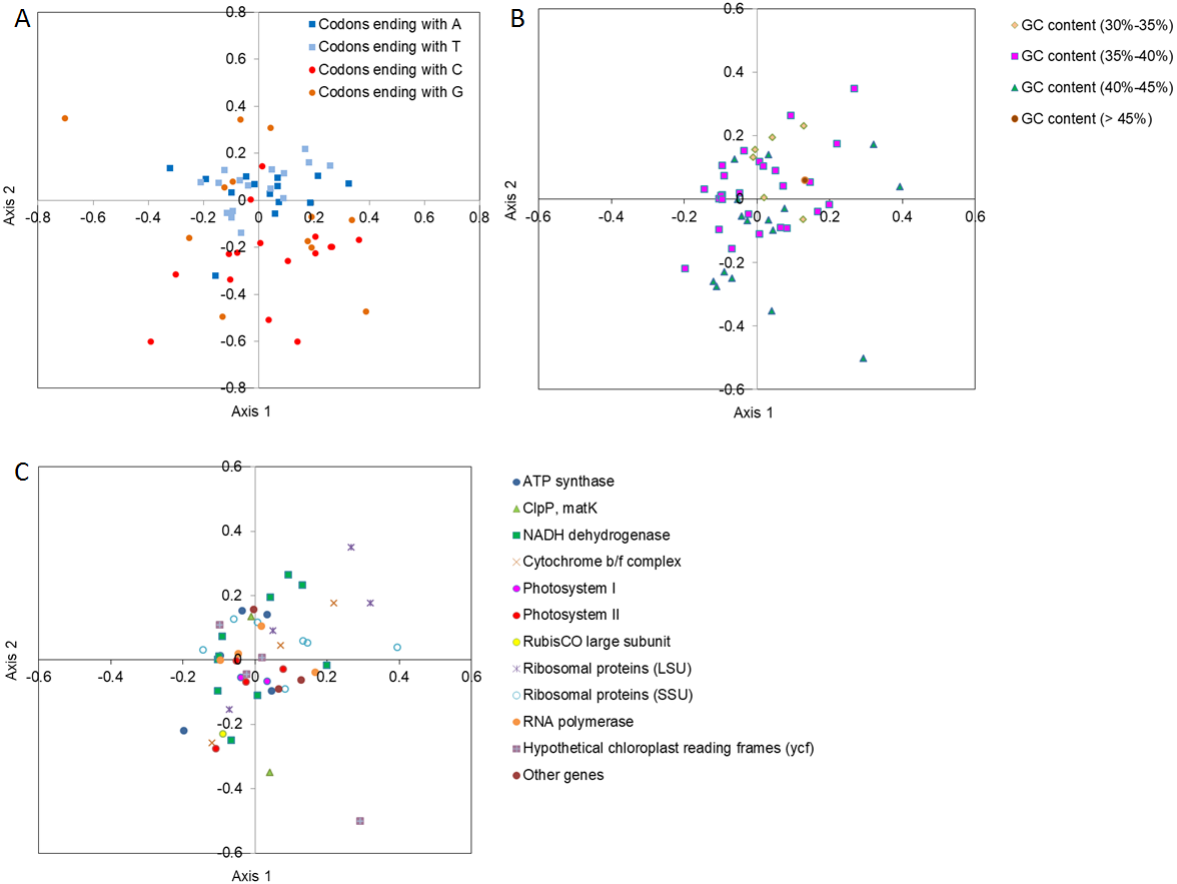
Correspondence analysis (COA) of SCU in *D. grandiflorum* cp genome. (A) COA analysis of SCU toward the codons, codons with different ending bases are represented by different colors. (B) COA analysis of SCU toward different GC contents, codons with different GC contents are represented by different colors. (C) COA analysis of SCU toward the coding genes, different gene types are represented by different colors and symbols.

To determine how the codons ending with different bases were contributing toward codon usage variation in the major axes of Axis 1 and Axis 2, the location of codons ending with different bases was drawn using different color points between Axis 1 and Axis 2 by COA analysis (Fig. 2A). The codons with A/T ends were closer to Axis 1 and were more tightly clustered than the codons with G/C ends, suggesting that the base composition probably affected the SCU bias. In contrast, the genes with lower GC contents (30%–35%) were distributed along the side of Axis 2, and the genes with a relatively lower GC content were more concentrated than the genes with a higher GC content (Fig. 2B), implying that GC content might influence the SCU bias. In addition, considering the positions of different functional gene groups, and following the direction along Axis 1 and Axis 2, we also found that the different groups were distributed discretely, indicating that many other factors (i.e., natural selection) might play a role in SCU bias (Fig. 2C).

In order to analyze the relationship of the important indices to the four main axes, correlation analysis was conducted to determine the central factors influencing codon usage bias (Table 4). The GC content showed an extremely negative correlation with Axis 2 (*P* <0.01), and the GC3s and GC3 contents also exhibited a significant negative correlation with Axis 2 and Axis 4.

**Table 4:** Correlation coefficients of the indices influencing codon bias in *D. grandiflorum* cp genome.

**Indices**	**GC**	**ENC**	**GC_3s_**	**GC_3_**	**Axis 1**	**Axis 2**	**Axis 3**	**Axis 4**
GC	1							
ENC	0.089	1						
GC3s	0.424^**^	0.548^**^	1					
GC3	0.437^**^	0.521^**^	0.964^**^	1				
Axis 1	0.123	0.275	0.198	0.166	1			
Axis 2	−0.393^**^	−0.498^**^	−0.623^**^	−0.664^**^	−0.004	1		
Axis 3	0.226	0.045	0.134	0.104	−0.002	0.005	1	
Axis 4	0.111	−0.331^*^	−0.385^**^	−0.292^*^	0.007	−0.015	−0.006	1

**Notes.**

* Positive correlation (*P* < 0.05).

** Significant positive correlation (*P* < 0.01).

GC G + C content of the gene ENC the effective number of codons GC3S the frequency of the nucleotides G + C at the 3rd of synonymous codons GC3 The G + C content at the 3rd of codons

### PR2-plot mapping analysis

**Figure 3:**
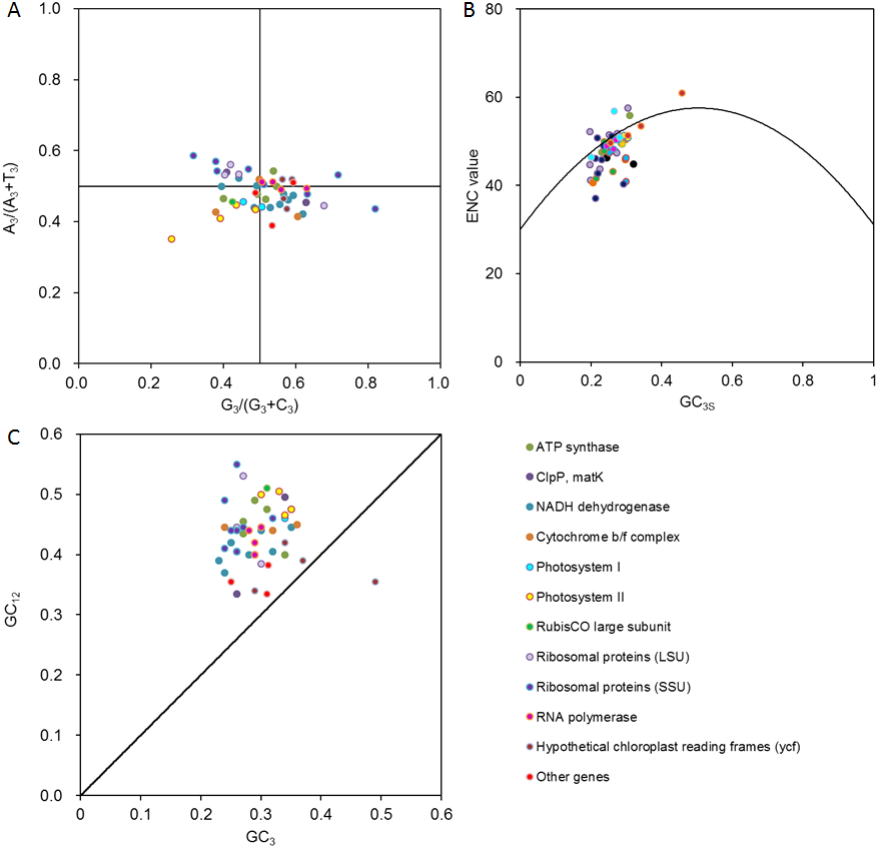
Multivariate statistical analysis for genes in *D. grandiflorum* cp genome. (A) PR2 analysis. A_3_/(A_3_ + T_3_): the ratio of A against A + T at the third position of codons. G_3_/(G_3_ + C_3_): the ratio of G against G + C at the third position of codons. The curves show the center line on 0.5. (B) ENC-plot analysis. ENC: effective number of codons. GC_3_s: the frequencies of nucleotide G + C at the third position of synonymous codons. The curve shows the expected relationship between ENC values and GC_3_s under random codon usage assumption. (C) Neutrality plot analysis. GC_12_: the average frequencies of nucleotide G + C at the first and second positions of synonymous codons. GC_3_: the frequencies of nucleotide G + C at the third position of synonymous codons. The curve shows that GC_12_ is equal to GC_3_.

Using PR2 plot mapping analysis, the points in our plot fell among 0.39 to 0.59 on A_3_/(A_3_ + T_3_), and 0.26 to 0.82 G_3_/(G_3_ + C_3_) (Fig. 3A). The genes were clearly distributed unevenly in the four quadrants centered on 0.5, with most points located under the horizontal centered line of 0.5 (in which the ratio of A_3_/(A_3_ + T_3_) <0.5) and a slightly greater number of points distributed on the right side of the vertical centered line of 0.5 (in which the ratio of G_3_/(G_3_ + C_3_) >0.5). These results indicated that the genes in *D. grandiflorum* preferred T and G, especially T at the third codon position.

**Figure 4:**
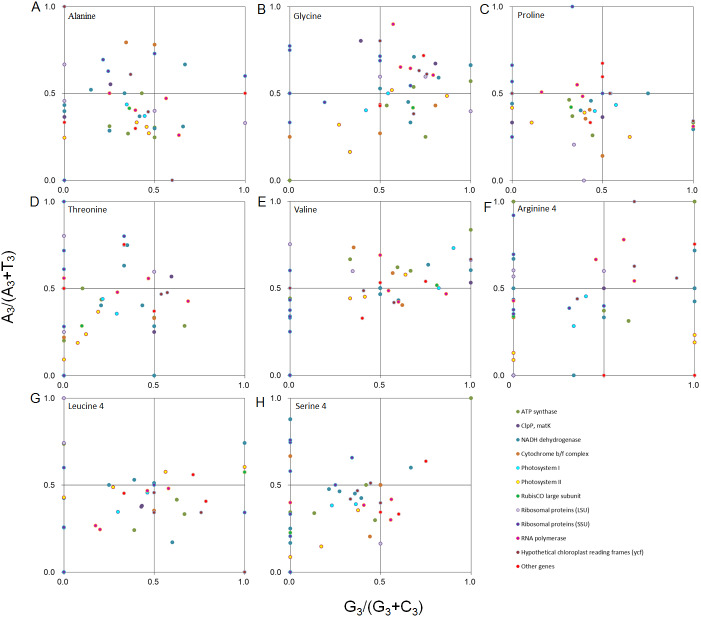
PR2 plot analysis of the four-degenerate synonymous-codon amino acids in *D. grandiflorum* cp genome. Alanine, glycine, proline, threonine, valine, arginine (CGA, CGU, CGG, and CGC), leucine (CUA, CUU, CUG, and CUC) and serine (UCA, UCU, UCG, and UCC) were shown in scatter diagrams (A–H), respectively. A_3_/(A_3_ + T_3_): the ratio of A against A + T at the third position of codons. G_3_ /(G_3_ + C_3_): the ratio of G against G + C at the third position of codons. The curves show the center line on 0.5.

Furthermore, we performed PR2 plot analysis of four-codon amino acids, including alanine, glycine, proline, threonine, valine, arginine (CGA, CGU, CGG, and CGC), leucine (CUA, CUU, CUG, and CUC), and serine (UCA, UCU, UCG, and UCC) (Fig. 4). It was clear that PR2 violation was the rule rather than the exception, and the distribution pattern was unique for each of the eight amino acids. The average value of A_3_/(A_3_ + T_3_) and G_3_/(G_3_ + C_3_) from the eight amino acids weighted with codon numbers for each gene was 0.44 and 0.38, respectively, suggesting that the eight amino acids had a preference for T and C when the eight amino acids were combined. Therefore, the balance between A/T and G/C was disrupted in *D. grandiflorum*.

### ENC plot analysis

An ENC plot was used to analyze the codon usage variation of the 51 CDSs in *D. grandiflorum* (Fig. 3B). Some genes were located on the standard curve toward the lower GC content region, for example, *rps3* and *rps4* from ribosomal proteins (SSU), *ndhI* and *ndhK* from NADH dehydrogenase, and so forth, which definitely originated from the extreme compositional constraints. However, a majority of the points were distributed away from the expected curve and were accompanied by a relatively concentrated distribution, suggesting that these genes should have additional codon usage biases, that are independent of compositional constraints. In addition, correlation analysis of the ENC and GC _3s_ values showed an extreme positive correlation (*r* = 0.548, *P*<0.01), suggesting that the base composition on the third position of the codons might play an important role in determining codon usage patterns.

### Neutrality plot analysis

From the neutrality plot, the relationship of GC_12_ and GC_3_ was analyzed, and the degree of change in natural selection and mutation pressure was estimated (Fig. 3C). The *ycf2* and *cemA* genes were located around the effected curve, while the remaining genes were above the standard curve. Using Pearson’s correlation analysis, a weak correlation of all coding genes between GC_12_ and GC_3_ was found (*r* = 0.261).

## Discussion

The transition of genetic information from mRNA to protein relies on the formation of codons (Chakraborty et al., 2017). The basic characteristic of a genetic code is that an amino acid is often encoded by different codon combinations, known as synonymous codons (Baeza et al., 2015). The uneven usage of synonymous codons with the same amino acid is reflected by SCU bias, and the SCU bias differs among various species and genes (Karumathil et al., 2018). The possible causes of SCU bias have been investigated in the genomes of numerous living organisms, for example, in *Zea mays, Arabidopsis thaliana*, cotton, and so others (Liu et al., 2010; Wang et al., 2018; Qiu et al., 2011). In this study, 51 CDSs of the *D. grandiflorum* cp genome were selected to analyze the SCU bias, and the possible factors influencing SCU bias were inferred.

### Unique codon usage pattern in the *D. grandiflorum* cp genome

The RSCU values reflect the codon usage pattern of different genes. The codon lacks bias when the RSCU value is less than 1 (Karumathil et al., 2018). In the *D. grandiflorum* cp genome, codons with the largest RSCU value based on each amino acid were suggested as high frequency codons. ENC reflects the degree of codon deviation from random selection and is an important index for reflecting the preference degree of the unequal use of synonymous codons (Gupta, Bhattacharyya & Ghosh, 2004). The range of ENC values is from 20 to 61, and the boundary value of ENC is 35. A value less than 35 represents strong codon preference, otherwise weak codon preference will occur (Song et al., 2018). In our study, the average ENC value of the codon genes was 48.12, implying a weak preference for SCU bias.

Our results indicated that the AT/GC nucleotide usage differed among the three positions of the codon, and these differences in base compositions might affect the total SCU bias in the *D. grandiflorum* cp genome. However, the overall SCU bias that we detected was low, which might be because the majority of codons were used during translation, and extreme SCU bias might only develop under particular conditions (Guan et al., 2018). In addition, we found that the genes from the *D. grandiflorum* cp genome showed a preference for AT-ending codons, particularly T-ending codons. Eight optimal codons further exhibited the similar patterns, seven of which ended with T, and one of which ended with A. PR2 is a rule of DNA base composition that endows A = T and G = C within a single strand when there is no any preference in mutation pressure and natural selection in both strands of DNA (Sueoka, 2001; Sueoka, 1995; Lobry, 1995). The present results showed that the distribution of genes with different ending bases was asymmetric and exhibited a preference for T-ending codons, and an apparent PR2 violation of the eight amino acids was further detected, thus revealing an SCU imbalance between A/T and G/C at the third base position. Our results were similar to those in other plant species. In the cotton genome, codons ending with T/A are preferred (Wang et al., 2018). A similar pattern was found in the codon usage of *Elaeagnus angustifolia* and *Porphyra umbilicalis* (Li et al., 2019; Wang et al., 2019). However, this phenomenon has not been observed in monocot species, for example, *Z. mays, Oryza sativa*, and *Hordium vulgare* (Liu et al., 2010; Wang & Hickey, 2007; Kawabe & Miyashita, 2003). The opposing patterns of codon ending bases might reflect the differences in differentiation between monocot and dicot plant species (Camiolo, Melito & Porceddu, 2015).

### Base composition affects the SCU bias of the *D. grandiflorum* cp genome

PCA analysis is usually used to analyze genes located in a 59-dimensional space and relies on the RSCU values. PCA can extract considerable variations and concentrate them together, thus helping to determine the major factors influencing SCU bias (Wei et al., 2014). In the present study, four main axes reflecting variation were determined, and the major indices versus the four axes were analyzed by correlation analysis. The codons with A/T endings plotted on Axis 1 and Axis 2 and showed a more tightly clustered distribution, indicating that this base composition could explain the variation in codon use. The significant correlations of GC content, GC_3s_ and GC_3_ content against the Axis 2 suggested that the base compositions as GC contents of the total and the third position of codons were valuable for SCU bias in the *D. grandiflorum* cp genome. However, Axis 1 and Axis 2 only explained 19.67% amount of the variation, and it appeared that the base composition had at most a partial influence on codon usage.

### Natural selection plays a major role in the SCU bias of *D. grandiflorum* cp genome

Synonymous codons are uneven by their nature, the mutations of which often occur at the 3rd base of a codon (Comeron & Aguadé, 1998). If there is no external pressure, as in the case of random mutation or mutation pressure in a certain direction, there should be no change in the three different positions of each codon and the base content should be similar (Guan et al., 2018). Thus, the preference for AT ends caused by directional substitution implied that evolutionary factors of SCU bias from *D. grandiflorum* cp genome were indeed existed. Generally, mutation pressure acts on nucleotide composition bias through shuffling A/T and G/C pairs, selection constraints lead to codon bias through maximizing protein production efficiency in high expressed genes (Guan et al., 2018). In our study, A/T and G/C at the third base position were asymmetric by PR2 analysis, and the significant correlations of GC content, GC_3s_ and GC_3_ content against the Axis 2 were found, which of them indicated that mutation pressure of base composition influenced SCU bias in the *D. grandiflorum* cp genome. However, Axis 2 only explained 8.96% amount of the variation, thus mutation pressure was not the determining factor shaping codon usage, other factors as well as natural selection might be more important than mutation pressure. ENC plot analysis and neutrality plot analysis are commonly combined to explore the two major evolutionary factors influencing codon usage in plant species (Wang & Hickey, 2007; Raubeson et al., 2007; Li et al., 2016). In order to determine whether natural selection was the main driving force affecting codon usage bias in the *D. grandiflorum* cp genome, we performed ENC plot analysis and neutrality plot analysis. ENC plot analysis is an important indicator that reflects the relationship of the two different indices (ENC value and GC_3s_), thus detecting the SCU variation among the genes (Wright, 1990). Wright concluded that the distribution comparison of genes and the standard curve could be indicative of some other factors, with the exception of mutation pressure. If the codon usage of a particular gene is under no selection, it should fall on the expected curve. In our study, it was observed that a few genes were positioned on the curve, which likely originated from the extreme mutation pressure. However, a majority of the points were lying well below the expected curve. This result suggested that a majority of genes in the *D. grandiflorum* cp genome had other SCU biases that were independent of mutation pressure, for example, natural selection. This hypothesis was largely supported by the neutrality plot mapping analysis. Neutrality plot analysis can effectively compare the effects of natural selection and mutation on codon usage bias (Sueoka, 1988). The low correlation between GC_12_ and GC_3_, that is, the smaller regression coefficient of approximately 0, showed that the base composition of the three positions differ, and the GC content of the cp genome is highly conserved, indicating that natural selection was the most important determinant of codon usage patterns. Conversely it shows that codon usage patterns are evidently reliant on mutation pressure (Zhang et al., 2018a; Zhang et al., 2018b; Zhang et al., 2018c). In the neutral graph, no correlation was found between GC_3_ and GC_12_, indicating a strong difference and that natural selection should be crucial for SCU bias in the *D. grandiflorum* cp genome. However, the signatures of selection constraints (positive, neutral, and negative) in *D. grandiflorum* cp genome could not be inferred for the lack of a reference sequence that is unaffected by selection, which need to be further detected in the following work.

## Conclusions

This study systematically analyzed the codon usage pattern in the *D. grandiflorum* cp genome, and the factors affecting SCU bias were comprehensively explored. The SCU bias in the *D. grandiflorum* cp genome is weak, preferring A/T ending bases. Excepting the notable mutation pressure effects, the majority of genetic evolution in the *D. grandiflorum* cp genome may be driven by natural selection. These results are the first to provide a clear set of SCU patterns and explore the possible evolutionary forces acting on the *D. grandiflorum* cp genome.

##  Supplemental Information

10.7717/peerj.10787/supp-1 Supplemental Information 1 Sequences of 117 genes in *D. grandiflorum.* cp genome

## Additional Information and Declarations

### Data Availability

The following information was supplied regarding data availability: The sequence information of genes are available as a Supplemental File. The complete chloroplast of *Delphinium grandiflorum* is available at Genbank: MN556604.
